# Understanding the Attitudes and Beliefs of Oncologists Regarding the Transitioning and Sharing of Survivorship Care

**DOI:** 10.3390/curroncol28060454

**Published:** 2021-12-19

**Authors:** Courtney H. Coschi, Daryl Bainbridge, Jonathan Sussman

**Affiliations:** 1 Department of Medicine, McMaster University, 1200 Main Street West, Hamilton, ON L8N 3Z5, Canada; coschic@hhsc.ca; 2 Juravinski Hospital and Cancer Centre, Department of Oncology, McMaster University, 711 Concession Street, Hamilton, ON L8V 1C3, Canada; bainbridgd@hhsc.ca; 3 Hamilton Health Sciences Juravinski Cancer Centre, 699 Concession Street, Hamilton, ON L8V 5C2, Canada

**Keywords:** survivorship, oncologist, primary care, models of care, shared care, transition

## Abstract

Transitioning survivorship care from oncologists to primary care physicians (PCPs) is a reasonable alternative to oncologist-led care. This study assessed oncologists’ attitudes and beliefs regarding sharing/transitioning survivorship care. A prospective survey of oncologists within a regional cancer program assessing self-reported barriers and facilitators to sharing/transitioning survivorship care was disseminated. In total, 63% (*n* = 39) of surveyed oncologists responded. Patient preference (89%) and anxiety (84%) are key to transition of care decisions; reduced remuneration (95%) and fewer longitudinal relationships (63%) do not contribute. Oncologists agreed that more patients could be shared/transitioned. Barriers include treatment-related toxicities (82% agree), tumor-specific factors (60–90% agree) and perception of PCP willingness to participate in survivorship care (47% agree). Oncologists appear willing to share/transition more survivors to PCPs, though barriers exist that warrant further study. Understanding these issues is critical to developing policies supporting comprehensive survivorship care models that address both cancer and non-cancer health needs. The demonstrated feasibility of this project warrants a larger-scale survey of oncologists with respect to the transition of survivorship care to PCPs, to further inform effective interventions to support high-quality survivorship care.

## 1. Introduction

Improvements in cancer screening and treatment have led to significant increases in the number of cancer survivors, each having a unique set of survivorship needs [1,2,3,4,5]. In Canada, survival for all cancers combined rose from 55% in the period 1992–1994 to 63% in the period 2012–2014 [1]. In 2015, the prevalence of cancer in Canadians was 2.1 million [6,7], and between 2015 and 2030, the number of new cancer cases diagnosed in Canada is expected to increase by 40% [8]. A significant proportion of cancer survivors consider their oncologists to be the main provider of comprehensive care after treatment, yet oncologists see their role as addressing cancer needs, and not other health conditions such as diabetes [7,9,10,11]. This leads to a potential gap in the quality of care. Patients who only see an oncologist are less likely to have their other comorbidities appropriately managed. For example, one study demonstrated that breast cancer survivors are more likely to die from modifiable conditions as compared to age and co-morbidity matched patients without breast cancer, and at the same time are less likely to receive chronic care for these other conditions [12]. There is accumulating evidence supporting increasing the involvement of primary care physicians (PCPs) in survivorship care. Studies have demonstrated that PCP involvement is associated with increased patient quality of life, better physical and emotional functioning, and greater patient empowerment over their own health care [13,14,15]. Further, when considering cancer-specific care, there appear to be no differences in recurrence-related events or time to detection of recurrence in published trials comparing oncology-led to primary care-led follow up care [16,17,18,19,20,21].

With increasingly limited resources within the cancer system in Canada, including lack of time and staff, there is a need to identify opportunities to provide optimal high-quality survivorship care in settings that include both the cancer treatment system and the community [11,22,23,24,25]. Health care systems similar to Canada, for example the United Kingdom and Australia, have also recognized the need to optimize the roles of providers in delivering cancer survivorship care [26,27]. Choosing Wisely Canada, a national voice for reducing health care system inefficiencies, has put forth the following recommendation “don’t deliver care (e.g., follow-up) in a high cost setting (e.g., inpatient, cancer centre) that could be delivered just as effectively in a lower-cost setting (e.g., primary care)” [28]. This recommendation applies to specialist follow-up care of cancer survivors that could otherwise be effectively managed by a patient’s PCP.

PCP and patient perspectives have been investigated with regard to transitioning from oncologist-led care to primary care [2,13,14,20,29,30,31,32,33,34,35]. Patients generally have confidence in PCPs to provide survivorship care, and support the use of a survivorship care plan (SCP) summarizing a patient’s cancer, cancer treatments, and recommended follow-up to facilitate this care [2,20,31]. Moreover, many PCPs are interested in assuming either shared or transitioned survivorship care for their patients, provided that they receive adequate support, including those surveyed in the same region in which oncologists were assessed in this study [20,30,31,36,37,38]. There is currently little information from the perspective of oncologists on how they view the delivery of survivorship care, enabling factors and those factors that act as barriers. Understanding these issues from the perspective of this key stakeholder group is critical to the design and implementation of comprehensive, whole person, survivorship care models that address both cancer and non-cancer health needs. Specifically, these insights would inform criteria to prompt consideration for transition of appropriate survivorship patients to primary care settings, and could ultimately support the development of provincial/national policies to support such models of survivorship care.

To address this knowledge gap, we conducted a prospective survey of oncologists within a regional cancer program to obtain a better understanding of their perceptions and practices of sharing care or transferring survivors back to primary care. Specifically, we assessed the frequency of respondents’ agreement and disagreement with barriers and facilitators to transition in association with predictor domains of interest including survivor demographics, health system factors, tumor- and treatment-specific factors, and toxicity profiles.

## 2. Materials and Methods

### 2.1. Design, Setting and Participants

A cross-sectional survey of all practicing medical, radiation, and hematologic oncologists, and general practitioner oncologists (GPOs) in a regional cancer centre was undertaken in Ontario, Canada. Health care in Ontario is a single payer health care universally funded system. The Juravinski Cancer Centre (JCC) is a regional cancer centre serving approximately 23,000 patients annually, 7500 of which are new patients. This results in over 200,000 visits per year. Ethics approval was obtained from the Hamilton Integrated Research Ethics Board.

### 2.2. Survey

A study-specific instrument was designed to assess current survivorship care practices of oncologists, as well as barriers and facilitators to models of transitioned or shared survivorship care with PCPs, as our review of the literature did not reveal an instrument to address our study-specific questions. Survivorship was defined as the stage of a patient’s experience with cancer where active therapy (ex. to induce remission) is complete, there is no evidence of active disease, and focus shifts to monitoring for recurrence, managing late and long-term effects of treatment, and general psychosocial health promotion. In order to collect data strategically so as to inform the development of interventions with a higher likelihood of applicability and success, we framed the development of the study-specific questionnaire on the updated 14-item theoretical domains framework (TDF) [39]. This framework identifies potential targets for health professional behavior change related to evidence-based practice [39,40]. Domains within the updated TDF applicable to this study include knowledge, skills, social/professional role and identity, beliefs about capabilities, beliefs about consequences, reinforcement, intentions and goals. A review of the relevant published literature in these domains, and expert opinion were used to optimize content validity [7,24,30,32,33,41,42,43].

Our first step involved a review of qualitative and quantitative instruments that contained similar questions on survivorship care from patient, PCP and oncologist perspectives published previously to ensure inclusion of important content [7,24,30,32,33,41,42,43]. Based on previously published data, four domains were identified for assessment: demographics, current practice, attitudes and beliefs, and barriers and facilitators. Within barriers and facilitators, questions were grouped by administrative, patient, personal and disease factors, as has been previously, and currently published. Expert opinion was sought with respect to the instruments’ coherence and comprehensiveness. The survey was pilot tested for readability, clarity, and content by one non-physician professional, one non-oncologist physician, and three practicing oncologists.

The finalized survey consists of twenty two questions, including multiple choice questions, Likert scales, and free text as follows: (1) demographics included practice setting, funding model, type of oncologist, years in practice, and disease sites treated (eight questions); oncologists were then asked to select one disease site in which to frame their responses to subsequent questions, (2) current practice (four multiple choice questions) and attitudes and beliefs (one matrix list of questions, and one Likert scale ranging from 1 = “strongly disagree” to 5 = “strongly agree”, and (3) barriers and facilitators in four domains (one Likert scale list of questions ranging from 1 = “strong barrier” to 5 = “strong facilitator” plus one free text for participants to list “other” barriers or facilitators, for each of the four domains). The disseminated survey is available as Supplementary File S1.

### 2.3. Data Collection

A web link to the online survey (hosted by SurveyMonkey) was e-mailed to all oncologists practicing at the JCC in November 2017. Weekly reminder e-mails were issued three times over four weeks, after which time the survey was closed. Collected data were imported into IBM SPSS version 24.

### 2.4. Statistical Analysis

Descriptive statistics were calculated for all demographic variables. Likert scales assessing attitudes and beliefs were divided into three categories: “disagree” (1–2), neither agree nor disagree (3), and “agree” (4–5). Likert scales assessing barriers and facilitators were divided into three categories: “barrier” (1–2), neither barrier nor facilitator (3), and “facilitator” (4–5) [44]. Free-text responses were assessed for frequency of themes using a constant comparison approach [45].

## 3. Results

### 3.1. Demographics

Out of a total of 62 oncology providers, 39 respondents completed the survey—a 63% response rate (Table 1). There was good representation across type of practicing oncologist. Missing data were negligible. The majority of respondents had been in practice for more than 11 years (67%). Only 5% of respondents were paid under a fee for service model, 39% were salaried and 44% reported a blended funding model. Respondents reported seeing a median of five new patients each week, ranging from two to 24 per week. There was broad representation across disease sites treated. The most commonly reported sites were breast (49%), other GI (44%), colorectal (26%) and lung (25%).

### 3.2. Current Self-Reported Practice

A total of 15% of oncologists reported that they do not transition any of their survivorship patients to PCPs. In contrast, 41% indicated that they currently transition up to 20% of these patients, and 20% of oncologists stated they transition more than 60% of their survivorship patients. These results did not appear to differ by respondent demographics, including disease site focus, although the sample was too small to rigorously analyze subgroup responses. The majority (72%) of oncologists reported that they generally start to consider transitioning their patients at or after five years under oncologist-led care; only 10% consider transition within the first year.

In thinking about their current survivor caseload (ex. survivorship patients under oncologist-led care), nearly all oncologists (92%) indicated that a proportion of these patients could reasonably be transitioned. Specifically, 24% of respondents stated that 21 to 40% could be transitioned, and 43% of respondents stated that more than 40% of their current survivorship patient population could be transitioned. Of the respondents who currently do not transition their survivorship patients, two thirds indicated that up to 60% of those patients could in fact be reasonably transitioned, whereas two respondents indicated that they still would not transition any survivorship patients to PCPs.

### 3.3. Attitudes and Beliefs

**Figure 1:**
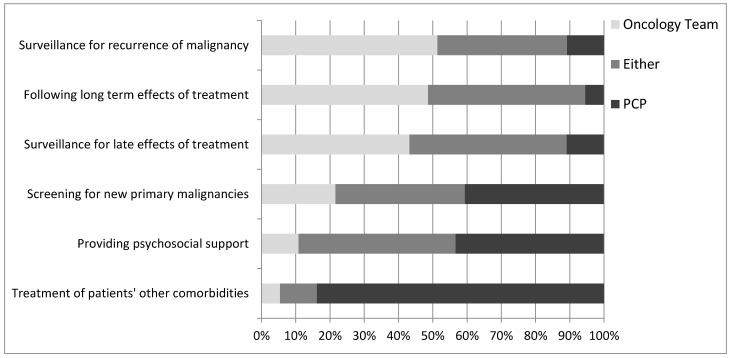
Oncologists’ opinions on who is the best to provide the indicated aspects of survivorship care.

Most respondents indicated that PCPs were best suited to treating patient’s non-cancer-related comorbidities (84%) (Figure 1). Opinions on the provision of psychosocial support were divided such that 43% of respondents felt that the PCP was best suited to provide this support, whereas about the same proportion (46%) felt that this could be managed by either oncologists or PCPs.

Almost half of respondents felt that oncologists were best suited to following the late (43%) and long-term (49%) effects of treatment, while 46% indicated that oncologists and PCPs were equally capable of following these effects. Slightly over half (51%) indicated that oncologists are best suited to follow patients for recurrence of malignancy, while 38% felt either the oncologist or PCP was capable. With regard to screening for new primary malignancies, 41% of respondents indicated that PCPs should be responsible, while 38% felt that either the oncologist or PCP could be responsible. These results did not appear to differ by respondent demographics, although sample size was too small for formal subgroup analysis.

**Figure 2:**
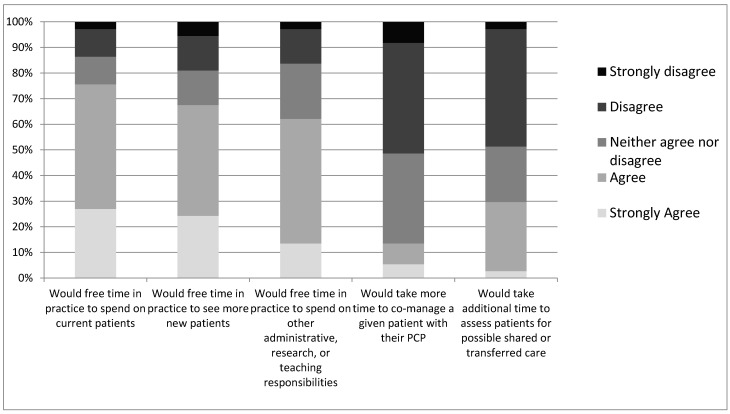
Oncologists’ level of disagreement or agreement on whether the indicated outcomes would change in their practice.

Oncologists were asked to speculate on the impact that transitioning more of their survivorship patients to PCPs could have on their practice (Figure 2). Of respondents, 67% of oncologists reported that it could free time in their practice to see more new patients (vs. 19% disagree), 76% percent reported that it could free time in their practice for current patients (vs. 13% disagree), and 30% agreed that it could take more time to assess patients for such transitioning of care (vs. 49% disagree). Finally, 63% of oncologists agreed that transitioning more of their survivorship patients would free up time to spend on administrative, research, teaching, or other responsibilities (vs. 16% disagree). For survivorship patients considered unsuitable for transition to PCPs, only 13% of respondents agreed that it would take more time to co-manage such a patient with PCPs (vs. 51% disagree).

### 3.4. Barriers and Facilitators

**Figure 3:**
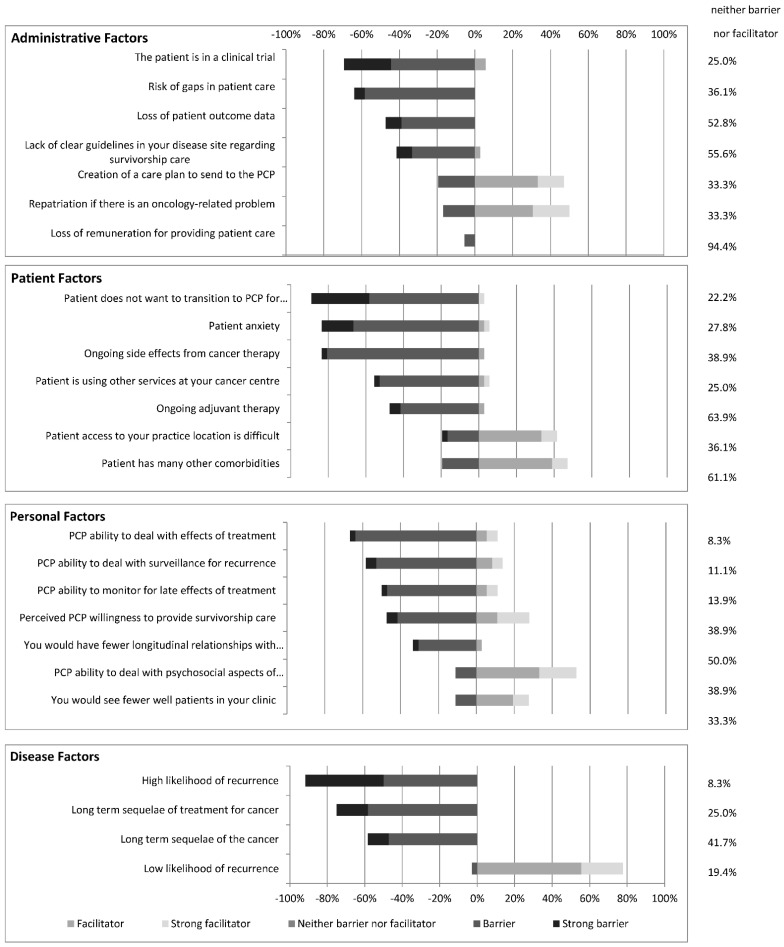
The number of oncologists who perceive the above administrative, patient, personal and disease factors as either barriers or facilitators to transitioning survivorship care to PCPs.

Oncologists were asked to rate the degree to which various administrative, personal, patient, and disease factors are barriers or facilitators to transitioning care of their survivorship patient population (Figure 3).

#### 3.4.1. Administrative Factors

Of the respondent oncologists, 94% indicated that loss of remuneration was neither a barrier nor facilitator to transitioning survivorship care (vs. 6% indicated this would be a barrier). Two more commonly perceived barriers were the perceived risk of gaps in patient care that may result from transitioning care (64%), and the patient being involved in a clinical trial (69%). As well, for any survivorship patient, 47% of oncologists indicated that the potential loss for patient outcome data is a barrier to transitioning care (vs. 53% neither a barrier nor facilitator).

Nearly half (47%) of oncologists indicated that providing a survivorship care plan to the PCP would facilitate transitioning survivorship care (vs. 19% indicated it would be a barrier). One half of oncologists (50%) also indicated that provided there was a clear path for patient repatriation for an oncology-related problem, it would facilitate transitioning survivorship care to PCPs. However, 42% of respondents felt that a lack of clear guidelines in their chosen disease site constituted a barrier to transitioning survivorship care (vs. 56% neither a barrier nor facilitator).

#### 3.4.2. Personal Factors

Only 11% of respondents indicated that the potential to see fewer well patients in clinic would be a barrier to transitioning survivorship patients (vs. 61% neither a barrier nor facilitator, and 23% facilitator). Similarly, only 33% indicated that having fewer longitudinal relationships with their patients would be a barrier (vs. 64% neither a barrier nor facilitator).

Next, the survey assessed oncologist’s perceptions of PCP ability and willingness to take on survivorship care of patients. Many oncologists felt that the PCP’s ability to deal with surveillance for recurrence (58%) or with the late and long-term effects of treatment (67%), as well as their willingness to provide survivorship care (47%) posed barriers to transitioning care. However, 53% of oncologists felt that a PCP’s ability to provide psychosocial support would facilitate transitioning care (vs. 36.1% neither a barrier nor facilitator).

#### 3.4.3. Patient Factors

Patient factors, as perceived by oncologists, that presented barriers to transitioning survivorship care included patient anxiety (83%), patient unwillingness to transition (89%), ongoing side effects from cancer therapy (83%), ongoing adjuvant therapy (47%), and patients using other services at the cancer centre where the oncologist practices—ex. social work, dietitian, pain clinic or psychologist (56%). Patient factors felt to facilitate transitioning survivorship care included patients having difficulty accessing the oncologist practice location (42%), and patients having multiple comorbidities (47%).

#### 3.4.4. Disease Factors

For the surveyed oncologists, a low likelihood of recurrence facilitated transitioning of survivorship care (78%), whereas a high likelihood of recurrence (92%), long-term sequelae of the cancer (58%), and long-term sequelae of the cancer treatment (75%) were seen as barriers to transitioning survivorship care to PCPs.

#### 3.4.5. Additional Factors

No additional factors were identified as facilitators or barriers to transitioning survivorship care through analysis of free-text responses.

## 4. Discussion

Although evidence suggests that, in many cases, care can transition safely from oncologists to PCPs, much survivorship care continues to be led by oncologists. Survivorship is a unique stage in a patient’s journey with cancer as in addition to important psychosocial support considerations, it can require both oncology-specific expertise as well as more generalized care of comorbid conditions, such as is provided by a patient’s PCP. For the purposes of our survey, survivorship was defined as the stage of a patient’s experience with cancer where active therapy (ex. to induce remission) is complete, there is no evidence of active disease, and focus shifts to monitoring for recurrence, managing late and long-term effects of treatment, and general psychosocial health promotion. It is clearly recognized that more survivorship care should be carried out by PCPs [32,46]. Our study builds on and complements previous work that was conducted with survivors and PCPs to explore Canadian oncologist’s perspectives on barriers and facilitators to adopting shared- or transitioned- survivorship care with PCPs.

In Canada, it is acknowledged that a new approach is required to improve care for cancer survivors [11,47]. This has also been clearly identified in other similar jurisdictions [26,27]. Moreover, with an increasing number of patients in the survivorship stage, there is also a need to provide optimal care with limited resources. Across provinces, it has been previously demonstrated that there are widely different patterns of specialist and PCP visits in the survivorship stage. For example, recent research found that in British Columbia, a much higher proportion of breast cancer survivors are followed exclusively by PCPs (33%) compared to the other provinces, ranging from 5% in Ontario to 16% in Nova Scotia [11]. The authors concluded that these differences across provinces are likely driven by differences in policies and initiatives, and variations in resources and infrastructure to support the transition to PCP-led follow-up care. This is supported by the fact that British Columbia (BC Cancer) in partnership with the Family Practice Oncology Network (FPON) offers a Cancer Care Outreach Program on Education (CCOPE), whose goal is to support PCPs in their growing role in cancer care, and to share best practices and resources through the cancer care continuum. Developing this capacity in PCPs has likely led to oncologists in British Columbia transitioning more of their cancer survivors to primary care for follow-up. 

In 2011, in Ontario, the Survivorship Program at Cancer Care Ontario (CCO) assessed follow-up programs at the 14 regional cancer centres in Ontario, and discovered a wide variety of follow-up care practices of breast cancer survivors across the province. This led CCO to fund each regional cancer centre in Ontario to develop new models of follow-up care for breast cancer survivors with the goal of implementing existing guidelines for breast cancer follow-up care [47]. Models of care that were developed included transition to PCPs, discharge to a transition clinic prior to discharge to PCPs, and shared care between the oncologist and PCPs. Each region developed a unique model of care, with all centres utilizing survivorship care plans, patient education materials, and a clear path for repatriation if recurrence was suspected. The study also found that funding to support personnel was likely essential to support changes in practice [47]. A plan to continue assessing the sustainability of these models by CCO was planned, as well as an assessment of ongoing patterns of care (ex. whether these developed models continued to be implemented). 

Despite evidence of success in other countries in the development of models for appropriate transitioning of survivors to community providers [7,27,48,49], in Canada, there largely remains a lack of routine sharing or transitioning of survivorship care with PCPs, as well as an absence of formal policies to help guide this practice. While patient and PCP perspectives on sharing and transitioning survivorship care have been explored as described above, there is a clear gap in understanding the specialist point of view. As demonstrated in other jurisdictions, it is important to understand the barriers and facilitators from the perspective of oncologists to support the development of models of survivorship care that include shared and transitioned care. Our study is the first in Canada within the last decade to explore the oncologist’s perspectives and self-reported practices of survivorship care [35,50,51,52]. Studies conducted prior were limited by either disease-type, oncologist-type, or method. A variety of facilitators and barriers to the sharing or transitioning of survivorship care with PCPs were identified across the studies but individually, studies were narrow in focus. For example, one study performed interviews of nine radiation and medical oncologists at a single centre. Another study surveyed medical oncologists treating breast cancer at a single centre, while one other study conducted nationally surveyed oncologists treating colorectal cancer [35,50,51,52]. Our study builds on this body of knowledge to date. We surveyed all types of oncologists from multiple disease sites to provide a descriptive summary of provider attitudes, and assessed barriers and facilitators to sharing or transitioning survivorship care that could be targeted for intervention.

### 4.1. Discrepancy between Oncologist Perceptions and PCP Willingness to Provide Survivorship Care

A number of previously reported studies have demonstrated that PCP involvement in survivorship care is associated with improved patient quality of life, better physical and emotional functioning, and greater patient empowerment over their own health care. There was also no demonstrated impact on recurrence detection or recurrence-related events [13,14,15,16,17,18,19,20,21]. As with other studies, we found that many of the oncologists we surveyed indicated that a significant proportion of their current survivorship population could reasonably be transitioned to PCPs [33,34]. Overall, 92% of respondents indicated a proportion of their survivorship patients could be considered for transitioned care. However, most of the respondents in our study perceived that PCPs may not be willing or able to take on the survivorship care of cancer patients, which has also be described previously by others [30,53]. This is at odds with perspectives of PCPs who have consistently identified that they are comfortable with managing cancer survivors and taking on a larger role in survivorship care. This comfort is contingent on having necessary supports and information readily available, such as through the use of survivorship care plans [20,30,31,36,37,38,54,55]. These findings offer a clear opportunity to design educational interventions directed at oncologists to address this perceptional barrier.

### 4.2. Facilitators and Barriers of Sharing/Transitioning Survivorship Care

Five key facilitators of transitioning survivorship care emerged from the oncologists’ responses. These are (i) the creation of a communication tool to support PCPs, (ii) an easy avenue for repatriation to oncologist-led care, (iii) PCP management of psychosocial aspects of survivorship care, (iv) patients having multiple other comorbidities, and (v) a low likelihood of malignancy recurrence.

Despite the fact that there are many facilitators to transitioning survivorship care, as well as willingness amongst responding oncologists, there are clearly barriers as reflected in the low self-reported rates. Our study identified four main barriers for providers practicing in our region: (i) administrative factors that included patient participation in a clinical trial, risk of gaps in patient care, loss of patient outcome data and lack of existing guidelines for disease sites; (ii) perceptions of PCP willingness and ability to provide survivorship care; (iii) patient factors such as patient anxiety, ongoing effects from cancer therapy, patient use of services at the cancer centre and ongoing adjuvant endocrine therapy; and (iv) disease with a high likelihood of recurrence, or long-term sequelae of the cancer or its treatment. In contrast to other studies, neither the time and effort to create SCPs, nor the loss of financial remuneration from sharing or transitioning survivorship patients were reported as barriers to sharing or transitioning care [56,57].

No novel facilitators or barriers were identified in this study, as evidenced by the lack of responses in free-text boxes provided in the survey.

### 4.3. Synthesis

Findings from our survey of oncologists practicing within a regional cancer program show that they are willing to share or transition survivorship care within the Canadian context. Our findings are consistent with previous work underscoring the importance of standardized transition communications using SCPs, repatriation pathways, and PCP involvement in psychosocial and co-morbid care [31,56,58,59,60,61,62,63] to support transitional care models. Although some emerging data suggest that SCPs may not improve patient-related outcomes, in our region and elsewhere, SCPs remain a widely accepted communication tool recognized by specialist physicians to support PCPs in the delivery of survivorship care [31,57]. SCPs are seen as an important tool to transfer clinical information between providers [31,33,54,58,59,60,61,62,63,64,65]. Specifically, SCPs facilitate clarifying provider roles in survivorship care, streamlining repatriation pathways to oncologist-led care, and informing appropriate surveillance for recurrence and management of late and long-term toxicities from treatment. Based on our survey responses and other studies, the use of SCPs directly addresses a number of the reported barriers to survivorship transition expressed by both oncologists and PCPs.

Finally, it appears that from oncologists’ perspectives, there are survivorship patient populations currently being followed that can be considered for transition. Our results highlight a need to more readily identify patients whose clinical course supports a shared or transitioned survivorship care approach. Further research into the disease- and treatment-specific concerns of oncologists could help to define low- and high-risk patient populations, thereby creating a framework to identify survivors whose care would best be served by sharing with or transitioning to PCPs. The policy implications of our findings include addressing the need for a systematic process for oncologists and primary care providers to personalize the model of follow up patient care based on a set of comprehensively developed, evidence informed criteria, that could be applied across broad populations of cancer survivors, as well as a minimum standard of SCPs to inform the transition. Barriers to be addressed from the cancer system include interventions to improve provider confidence in community management of treatment side effects, real-time data on outcomes, and clear pathways to repatriate transitioned patients for assessment when needed.

### 4.4. Strengths and Limitations

A number of limitations need to be considered in the interpretation of our findings. The self-reported nature of the survey data may be prone to satisficing, and not truly represent respondents’ current practices. As well, there may have been differences based on provider characteristics that we were unable to identify, given the overall number of respondents was too small to conduct subgroup analyses. The absence of responses to the “other facilitators or barriers” open-text questions may indicate that respondents felt no need to reflect further on additional factors, rather than that these factors had already been exhaustively covered in the survey. Finally, the opinions collected in these surveys were collected from a single, regional cancer centre, and therefore may or may not be representative of the opinions of oncologists in other jurisdictions.

One of the strengths of this study was that the survey was constructed based on review of the relevant literature and consideration of previous surveys of oncologists in Canada, the United States and Europe to ensure inclusion of important theoretical constructs. The comprehensiveness of the questionnaire is demonstrated in that no other barriers, facilitators, or other concerns clearly emerged from the open text comments that were not otherwise addressed in the survey. We received a good response rate (63%) from a broad sample of oncologists, practicing in a variety of disease sites, though the opinions of non-respondents may differ from the opinions captured. 

## 5. Conclusions

Our study found that oncologists clearly identified that a substantial proportion of their survivorship patient population could be shared with or transitioned to PCPs. Oncologist perceptions are integral to applying knowledge-translation strategies, and developing future policy for survivorship care. The compelling facilitators to non-oncologist-led survivorship care still appear to be outweighed by barriers. Interventions to address identified barriers could include strategies to better educate oncologists that PCPs are both willing and able to take on survivorship care of cancer patients, development of easy to use SCPs to share with PCPs and creating a more systematic process for oncologists to identify patients who are suitable for the transition of survivorship care to PCPs. In addition to addressing oncologist-identified barriers, PCPs and other health providers including nurses must be included in the design of new models of care to best optimize the delivery of cancer survivorship care.

It is important to extend our survey to other regions to expand the generalizability of the study findings. The demonstrated feasibility of this project supports larger-scale surveys of oncologists in other Canadian and international jurisdictions, to further inform optimal delivery of cancer survivorship care within the context of these care systems.

## Figures and Tables

**Table 1:** Characteristics of respondents (*N* = 39).

Characteristic			No.	%
**New patients/week**		Median = 5, range = 2–24		
**Funding Model**		Blended	17	43.6
		Salary	15	38.5
		Fee for service	2	5.1
		No response	5	12.8
**Years in practice**		≤5	5	12.8
		6–10	8	20.5
		11+	26	66.7
**Main area of clinical practice**				
	Radiation oncology		18	46.2
	Medical oncology		12	30.8
	Hematology/oncology		4	10.3
	Gynecologic		1	2.6
	General medicine		4	10.3
**Type(s) of cancer treated on regular basis ^a^**				
	Breast		19	48.7
	Other gastrointestinal		17	43.6
	Colorectal		10	25.6
	Lung		8	20.5
	Prostate		5	12.8
	Hematologic		5	12.8
	Sarcoma		5	12.8
	Central nervous system		5	12.8
	Skin		4	10.3
	Head and Neck		4	10.3
	Non-prostate genitourinary		4	10.3
	Gynecologic		2	5.1
	Other		10	25.6
**Considered primary cancer site ^b^**				
	Breast		10	25.6
	Hematologic		5	12.8
	Lung		5	12.8
	Other gastrointestinal		4	10.3
	Central nervous system		3	7.7
	Prostate		3	7.7
	Colorectal		2	5.1
	Gynecologic		2	5.1
	Skin		2	5.1
	Head and neck		1	2.6
	Sarcoma		1	2.6
	Other		1	2.6

**^a^** Multiple responses are possible. **^b^** Disease site used to frame subsequent survey responses.

## Data Availability

The data presented in this study are available on reasonable request from the corresponding author.

## Supplementary Materials

The following are available online at https://www.mdpi.com/article/10.3390/curroncol28060454/s1, File S1: Disseminated Survey.
