# Supplementary material for: Understanding the Attitudes and Beliefs of Oncologists Regarding the Transitioning and Sharing of Survivorship Care
Source: Curr Oncol. 2021 Dec 19;28(6):5452–65. doi: 10.3390/curroncol28060454 (PMC8700375; doi:10.3390/curroncol28060454)
Supplement: Supplementary file 1 [file curroncol-28-00454-s001.zip › curroncol-1474615-supplementary.pdf]

Question:

This survey is designed to assess the following: of oncologists practicing at the Juravinski and Walker Family Cancer Centres, 1) how many oncologists are engaging in transitioning/sharing survivorship care with Primary Care Physicians (PCPs), 2) what are the attitudes and beliefs of oncologists regarding transitioning/sharing survivorship care with PCPs, and 3) what are any personal, patient, malignancy-specific and administrative barriers and/or facilitators to the transition/sharing of survivorship care with PCPs?

Definitions:

Primary Care Physician (PCP): the physician providing primary health care to your patient (ex. Family Physician).

Survivorship: the stage of a patient's experience with cancer where active therapy (ex. to induce remission) is complete, there is no evidence of active disease, and focus shifts to monitoring for recurrence, managing late and long term effects of treatment, general and psychosocial health promotion.

Sharing care: survivorship care for the patient is split between the oncologist and the PCP, assuming clear roles are delineated.

Transitioning care: survivorship care is solely under the purview of the PCP.

Survey

Demographic and Practice Features:

1. Type of Oncologist (check one):
   - ◯ Medical
   - ◯ Radiation
   - ◯ GPO/family physician + oncology
   - ◯ Hematologist
   - ◯ Surgical (including gynecologic oncologist)
   - ◯ Other

2. Practice setting (check one):
   - ◯ Regional Cancer Centre
   - ◯ Non-Regional Cancer Centre
   - ◯ Community Hospital

3. Oncologist funding model:
   - ◯ Fee-for service
   - ◯ Alternate Funding Program (AFP)
   - ◯ Blended model

4. Average # of new patients/week: ____________

5. Years in practice:
   ◯ ≤ 5
   ◯ 6-10
   ◯ 11-15
   ◯ 16-20
   ◯ >20

6. What percent of your professional time do you spend on the following activities in a week:

Providing patient care (including radiation planning sessions, attending multi-disciplinary rounds, attending talks)	________	%
Research	________	%
Teaching	________	%
Administrative duties	________	%
Other (specify)	________	%

7. Disease site(s) in your current practice (check all that apply):
   ◯ Breast
   ◯ Colon
   ◯ Skin
   ◯ Prostate
   ◯ Hematologic
   ◯ Sarcoma
   ◯ Other
   ◯ CNS
   ◯ GI
   ◯ Head and neck
   ◯ Non-prostate GU
   ◯ Gynecologic
   ◯ Lung

8. If you treat more than once disease site, please choose one, and answer the rest of the survey for this disease site only. Indicate chosen disease site:
   ◯ Breast
   ◯ Colon
   ◯ Skin
   ◯ Prostate
   ◯ Hematologic
   ◯ Sarcoma
   ◯ Other
   ◯ CNS
   ◯ GI
   ◯ Head and neck
   ◯ Non-prostate GU
   ◯ Gynecologic
   ◯ Lung

Attitudes and Beliefs:

1. From your clinical experience for the disease site you treat most often, in your opinion, who is best suited to provide the following survivorship care activities:

Following long term effects of treatment	◯ Oncology team	◯ PCP	◯ Either
Surveillance for late effects of treatment	◯ Oncology team	◯ PCP	◯ Either
Screening for new primary malignancies	◯ Oncology team	◯ PCP	◯ Either
Surveillance for recurrence of malignancy	◯ Oncology team	◯ PCP	◯ Either
Providing psychosocial support	◯ Oncology team	◯ PCP	◯ Either
Treatment of patient's other co-morbidities	◯ Oncology team	◯ PCP	◯ Either
Other (Please specify: __________________)	◯ Oncology team	◯ PCP	◯ Either

2. In thinking about transitioning/sharing survivorship care with PCPs, please indicate the degree to which you think the following aspects of your practice would be affected:

It would free time in your practice to spend on current patients.

1	2	3	4	5
Strongly Disagree		Neither agree nor disagree		Strongly Agree

It would free time in your practice to spend on other administrative, research, or teaching responsibilities.

1	2	3	4	5
Strongly Disagree		Neither agree nor disagree		Strongly Agree

It would take additional time to assess patients for possible shared or transferred care.

1	2	3	4	5
Strongly Disagree		Neither agree nor disagree		Strongly Agree

It would take more time to co-manage a given patient with their PCP.

1	2	3	4	5
Strongly Disagree		Neither agree nor disagree		Strongly Agree

It would free time in your practice to see more new patients.

1	2	3	4	5
Strongly Disagree		Neither agree nor disagree		Strongly Agree

Other. Specify: ____.

1	2	3	4	5
Strongly Disagree		Neither agree nor disagree		Strongly Agree

Other. Specify: ____.

1	2	3	4	5
Strongly Disagree		Neither agree nor disagree		Strongly Agree

3. What proportion of your survivorship patient population (ex. no evidence of active disease), do you currently transition to PCPs:
   - ◯ None
   - ◯ 1-10%
   - ◯ 11-20%
   - ◯ 21-30%
   - ◯ 31-40%
   - ◯ ≥41%

4. At what point in your patient's survivorship stage do you generally consider transition to PCPs:
   - ◯ <1 year
   - ◯ 1 year
   - ◯ 2 years
   - ◯ 3 years
   - ◯ 4 years
   - ◯ 5 years
   - ◯ >5 years

5. If you think about your current survivorship patient population (ex. no evidence of active disease), you feel the following proportion could be reasonably *transitioned* to PCPs:
   - ◯ None
   - ◯ 1-10%
   - ◯ 11-20%
   - ◯ 21-30%
   - ◯ 31-40%
   - ◯ ≥41%

6. If you think about your current survivorship patient population (ex. no evidence of active disease), you feel the following proportion could be reasonably *shared* with PCPs:
   - ◯ None
   - ◯ 1-10%

◯ 11-20%
◯ 21-30%
◯ 31-40%
◯ ≥41%

<u>Assessment of Barriers and Facilitators:</u>

Barrier: that which you perceive to impede *<u>your</u>* decision to transition patient care to a PCP
Facilitator: that which you perceive to enable *<u>your</u>* decision to transition patient care to a PCP

To what degree would the following *<u>administrative factors</u>* be barriers or facilitators to transitioning/sharing survivorship care:

Loss of remuneration for providing patient care.

1	2	3	4	5
◯	◯	◯	◯	◯
Strong Barrier		Neither barrier nor facilitator		Strong Facilitator

Creation of a care plan to send to the PCP.

1	2	3	4	5
◯	◯	◯	◯	◯
Strong Barrier		Neither barrier nor facilitator		Strong Facilitator

Repatriation if there is an oncology-related problem.

1	2	3	4	5
◯	◯	◯	◯	◯
Strong Barrier		Neither barrier nor facilitator		Strong Facilitator

The patient is in a clinical trial.

1	2	3	4	5
◯	◯	◯	◯	◯
Strong Barrier		Neither barrier nor facilitator		Strong Facilitator

Risk of gaps in patient care.

1	2	3	4	5
◯	◯	◯	◯	◯
Strong Barrier		Neither barrier nor facilitator		Strong Facilitator

Loss of patient outcome data.

1	2	3	4	5
Strong Barrier		Neither barrier nor facilitator		Strong Facilitator

Lack of clear guidelines in your disease site regarding survivorship care.

1	2	3	4	5
Strong Barrier		Neither barrier nor facilitator		Strong Facilitator

Other. Specify: ____.

1	2	3	4	5
Strong Barrier		Neither barrier nor facilitator		Strong Facilitator

Other. Specify: ____.

1	2	3	4	5
Strong Barrier		Neither barrier nor facilitator		Strong Facilitator

To what degree would the following *personal factors* be barriers or facilitators to transitioning/sharing survivorship care:

Barrier: that which you perceive to impede *your* decision to transition patient care to a PCP
Facilitator: that which you perceive to enable *your* decision to transition patient care to a PCP

You would see fewer well patients in your clinic.

1	2	3	4	5
Strong Barrier		Neither barrier nor facilitator		Strong Facilitator

You would have fewer longitudinal relationships with patients.

1	2	3	4	5
Strong Barrier		Neither barrier nor facilitator		Strong Facilitator

PCP ability to deal with surveillance for recurrence.

1	2	3	4	5
Strong Barrier		Neither barrier nor facilitator		Strong Facilitator

PCP ability to deal with effects of treatment.

1	2	3	4	5
Strong Barrier		Neither barrier nor facilitator		Strong Facilitator

PCP ability to monitor for late effects of treatment.

1	2	3	4	5
Strong Barrier		Neither barrier nor facilitator		Strong Facilitator

PCP ability to deal with psychosocial aspects of survivorship care.

1	2	3	4	5
Strong Barrier		Neither barrier nor facilitator		Strong Facilitator

Perceived PCP willingness to provide survivorship care.

1	2	3	4	5
Strong Barrier		Neither barrier nor facilitator		Strong Facilitator

Other. Specify: ____.

1	2	3	4	5
Strong Barrier		Neither barrier nor facilitator		Strong Facilitator

Other. Specify: ____.

1	2	3	4	5
Strong Barrier		Neither barrier nor facilitator		Strong Facilitator

To what degree are the following *patient factors* barriers or facilitators to transitioning/sharing survivorship care:

Barrier: that which you perceive to impede *your* decision to transition patient care to a PCP
Facilitator: that which you perceive to enable *your* decision to transition patient care to a PCP

Patient anxiety.

1	2	3	4	5
Strong Barrier		Neither barrier nor facilitator		Strong Facilitator

Patient access to your practice location is difficult.

1	2	3	4	5
Strong Barrier		Neither barrier nor facilitator		Strong Facilitator

Ongoing adjuvant therapy (ex. endocrine therapy in breast cancer).

1	2	3	4	5
Strong Barrier		Neither barrier nor facilitator		Strong Facilitator

Ongoing side effects from cancer therapy.

1	2	3	4	5
Strong Barrier		Neither barrier nor facilitator		Strong Facilitator

Patient has many other co-morbidities.

1	2	3	4	5
Strong Barrier		Neither barrier nor facilitator		Strong Facilitator

Patient is using other services at your cancer centre (ex. psychologist, social work, dietician, pain clinic, other oncology physicians).

1	2	3	4	5
Strong Barrier		Neither barrier nor facilitator		Strong Facilitator

Patient does not want to transition to PCP for malignancy-related care.

1	2	3	4	5
Strong Barrier		Neither barrier nor facilitator		Strong Facilitator

Other. Specify: ____.

1	2	3	4	5
Strong Barrier		Neither barrier nor facilitator		Strong Facilitator

Other. Specify: ____.

1	2	3	4	5
Strong Barrier		Neither barrier nor facilitator		Strong Facilitator

To what degree are the following *__disease factors__* barriers or facilitators to transitioning/sharing survivorship care:

Barrier: that which you perceive to impede *__your__* decision to transition patient care to a PCP
Facilitator: that which you perceive to enable *__your__* decision to transition patient care to a PCP

Low likelihood of recurrence.

1	2	3	4	5
Strong Barrier		Neither barrier nor facilitator		Strong Facilitator

High likelihood of recurrence.

1	2	3	4	5
Strong Barrier		Neither barrier nor facilitator		Strong Facilitator

Long term sequelae of the cancer.

1	2	3	4	5
Strong Barrier		Neither barrier nor facilitator		Strong Facilitator

Long term sequelae of treatment for cancer.

1	2	3	4	5
Strong Barrier		Neither barrier nor facilitator		Strong Facilitator

Other. Specify: ____.

1	2	3	4	5
Strong Barrier		Neither barrier nor facilitator		Strong Facilitator

Other. Specify: ____.

1	2	3	4	5
Strong Barrier		Neither barrier nor facilitator		Strong Facilitator

7. For the disease sites that you treat, during a patient's survivorship stage, is there any point at which you would be comfortable with transitioning to PCP-led care? Please answer this question for each disease site you treat.
   ◯ Yes (When? ___________________________________)
   ◯ No (Why not? _________________________________)

8. Thank you for your time. We would appreciate any further feedback you have with regards to improving survivorship care. Please feel free to comment on any other issues, or other barriers or facilitators, which you feel are important, especially if you feel they have not been adequately covered by the previous survey questions.
   Specify. ___________________________________

9. If you are interested in being contacted to be interviewed on the topics addressed in this survey, please click on this link. This link will bring you to a page where you can provide contact information that is separate from your survey responses. Thank you again for your time.
